# Haspin-dependent and independent effects of the kinase inhibitor 5-Iodotubercidin on self-renewal and differentiation

**DOI:** 10.1038/s41598-019-54350-4

**Published:** 2020-01-14

**Authors:** Eleftheria Karanika, Katerina Soupsana, Anastasia Christogianni, Dimitris Stellas, Apostolos Klinakis, Anastasia S. Politou, Spyros Georgatos

**Affiliations:** 1 Stem Cell and Chromatin Group, Institute of Molecular Biology and Biotechnology, Biomedical Division, FORTH-ITE, Ioannina, Greece; 2 Laboratory of Biology, University of Ioannina, Faculty of Medicine, Ioannina, Greece; 3 Laboratory of Biological Chemistry, University of Ioannina, Faculty of Medicine, Ioannina, Greece; 4 Biomedical Research Foundation, Academy of Athens, Athens, Greece; 5 Present Address: Human Retrovirus Pathogenesis Section, Vaccine Branch, Center for Cancer Research, National Cancer Institute at Frederick, Frederick, USA

**Keywords:** Cell biology, Phosphorylation, Molecular biology

## Abstract

The kinase Haspin phosphorylates histone H3 at threonine-3 (H3T3ph), creating a docking site for the Chromosomal Passenger Complex (CPC). CPC plays a pivotal role in preventing chromosome misalignment. Here, we have examined the effects of 5-Iodotubercidin (5-ITu), a commonly used Haspin inhibitor, on self-renewal and differentiation of mouse embryonic stem cells (ESCs). Treatment with low concentrations of 5-ITu eliminates the H3T3ph mark during mitosis, but does not affect the mode or the outcome of self-renewal divisions. Interestingly, 5-ITu causes sustained accumulation of p53, increases markedly the expression of histone genes and results in reversible upregulation of the pluripotency factor Klf4. However, the properties of 5-ITu treated cells are distinct from those observed in Haspin-knockout cells generated by CRISPR/Cas9 genome editing, suggesting “off-target” effects. Continuous exposure to 5-ITu allows modest expansion of the ESC population and growth of embryoid bodies, but release from the drug after an initial treatment aborts embryoid body or teratoma formation. The data reveal an unusual robustness of ESCs against mitotic perturbants and suggest that the lack of H3T3ph and the “off-target” effects of 5-ITu can be partially compensated by changes in expression program or accumulation of suppressor mutations.

## Introduction

The metazoan genomes encode >500 typical, atypical and pseudo- protein kinases^1,2^. Of these, about 100 are directly or indirectly involved in cell division^3,4^. One kinase that appears to play important role in mitosis is Haspin, a protein originally identified in mouse testes and now known to be expressed in all somatic cells^5–8^. It is encoded by a single-copy gene (*Gsg2*) and is highly conserved in eukaryotic organisms, from humans to yeast^9^.

Haspin possesses distinct N-terminal and C-terminal domains. The C-terminal domain (residues 452–789 in the human protein) folds autonomously as a bilobar structure and has measurable kinase activity. However, by comparison to other eukaryotic protein kinases, Haspin^452–789^ seems to possess atypical features: an α-helical extension preceding the G-loop and two insertions further downstream bury the N-terminal lobe almost entirely; the A-loop is shorter than usual and starts with a DYT, instead of a DFG, motif; there is an additional insertion between the β7 and the β8 loops; and finally, a deletion in the C-terminal lobe removes the αG helix, which is conspicuous in other members of the kinase superfamily^10,11^.

Despite these structural differences, Haspin^452–789^ phosphorylates specifically the N-terminal “tail” of histone H3 at Thr3 (H3T3ph)^10,11^. In somatic cells, H3T3ph is detected at the centromeric area of mitotic chromosomes^12–14^ and serves as a binding site for Survivin, a subunit of the Chromosomal Passenger Complex (CPC)^15–17^. CPC deposits the master kinase Aurora B near the sites of microtubule-chromosome contact, allowing correction of improper attachments and activation of the Spindle Assembly Checkpoint (SAC) in the event of chromosome misalignment^18–21^.

Functional analysis of Haspin has been facilitated by the availability of small molecule inhibitors. One such compound is 5-Iodotubercidin (5-ITu), an adenosine derivative. 5-ITu was initially characterized as an adenosine kinase inhibitor^22,23^, but later found to inhibit Haspin *in vitro*^11,24,25^. At concentrations from 0.5 to 1.0 μM, 5-ITu abolishes the H3T3ph mark in living cells, but does not affect the kinases Aurora A, Aurora B, Bub1, Cdk1/Cyclin B, Mps1, Plk1 and Nek2A^25–27^. For this reason, it has been used as a tool for investigating the role of Haspin in a variety of cellular systems^26–29^.

Here, we have employed 5-ITu to study the role of Haspin-mediated phosphorylation during self-renewal and differentiation of mouse embryonic stem cells. We have found that treatment with low concentrations of 5-ITu eliminates the H3T3ph mark during mitosis, but does not affect the mode or the final outcome of self-renewal divisions. However, 5-ITu changes the expression pattern of numerous genes and has unexpected, “off-target” effects on stem cell growth and differentiation. We discuss below these results, distinguishing between Haspin-dependent and Haspin independent effects of 5-ITu and arguing about the mechanisms that allow completion of cell division in the absence of H3T3ph.

## Results

### Effects on division mode

**Figure 1:**
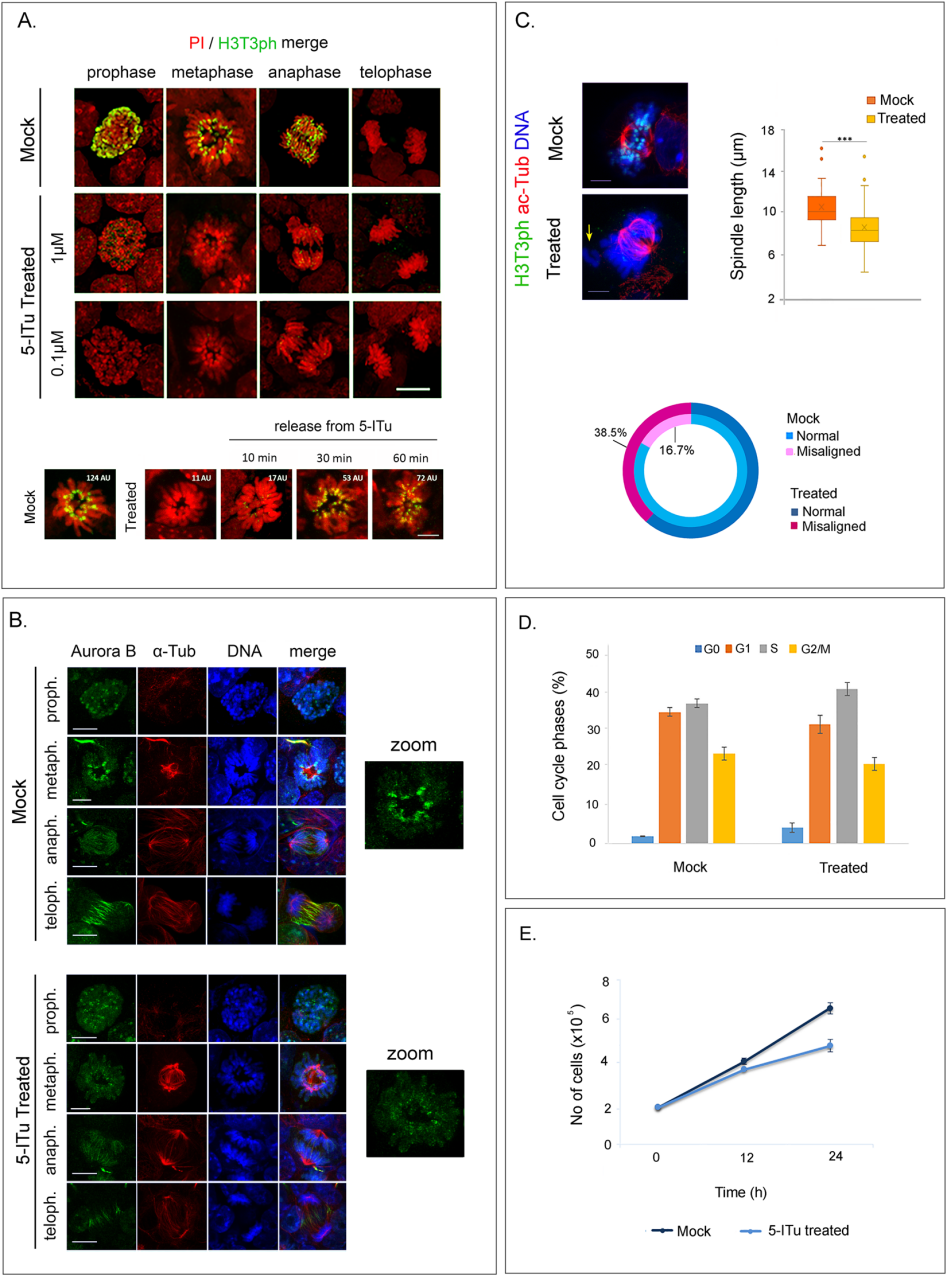
Effects of 5-ITu on self-renewal divisions. (**A**) Detection of H3T3ph in different phases of mitosis after treatment of E14 cells with 5-ITu (0.1 μΜ/1.0 μΜ, 90 min). Notice that the H3T3ph signal disappears after incubation with the Haspin inhibitor, but re-appears when the agent is removed from the media. The specimens shown are counter-stained with propidium iodide (PI). Bar: 5 μm. **(B)** Aurora B distribution in cells treated with 5-ITu (1.0 μΜ, 26 h). The samples are doubly stained with anti-α-tubulin antibodies and counter-stained with TOPRO-3. The cutouts on the right depict metaphase profiles at higher zoom. Bar: 5 μm. **(C)** Detection of spindle length differences and chromosome misalignment defects in 5-ITu treated cells (1.0 μΜ, 26 h). The images show profiles of metaphase cells stained with anti-H3T3ph and anti-Tubulin antibodies. The samples are counterstained with TOPRO-3. Bar:5 μm. (see also Fig. S1A) **(D**) Distribution of treated and untreated cells in the various phases of cell cycle as detected by flow cytometry (1.0 μΜ, 26 h). (**E**) Growth curves of treated and untreated cells (1.0 μΜ, 26 h). “Mock” implies treatment with solvent alone (EtOH).

In a first series of experiments, we used indirect immunofluorescence microscopy to examine the effects of 5-ITu on self-renewal divisions of E14 cells. As shown in Fig. 1A, exposure to 0.1–1.0μΜ 5-ITu for 90 min eliminated the H3T3ph signal in all mitotic cells. This effect was reversible: after treatment with 1.0μΜ 5-ITu for 26 h (roughly the duration of two cell cycles) and subsequent removal of the drug from the media, H3T3ph re-emerged within 30–60 min. In the absence of H3T3ph, CPC targeting to the centromere was less efficient, as could be inferred by the re-distribution of Aurora B from the centromeric area to the arms of the mitotic chromosomes (Figs. 1B and S2). In addition, the length of the mitotic spindle became slightly shorter and the proportion of cells with misaligned chromosomes increased (Fig. 1C).

**Figure 2:**
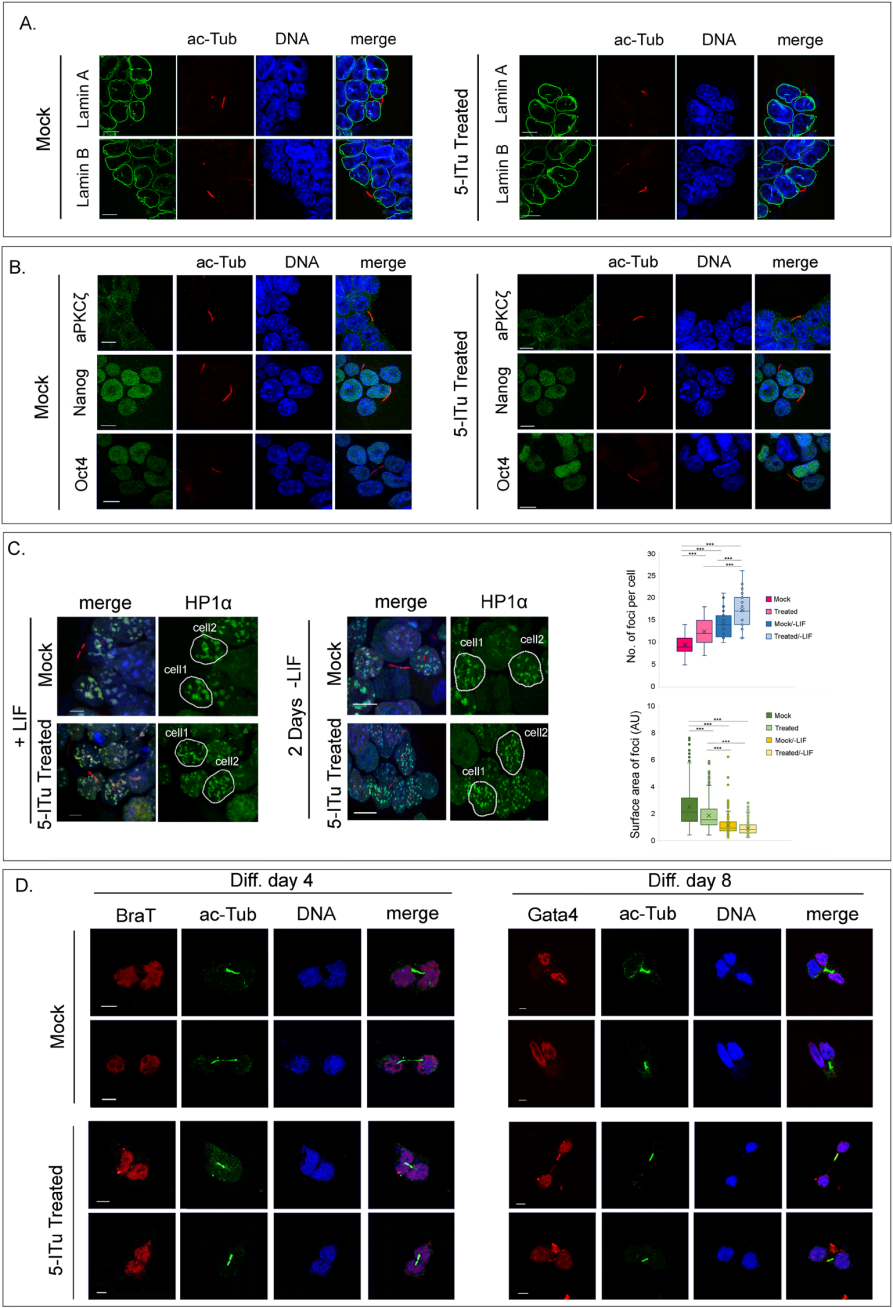
Organization of the post-mitotic nucleus and distribution of lineage markers in 5-ITu treated cells (1.0 μΜ, 26 h). (**A**) Lamin A and lamin B localization in cells treated with 5-ITu (1.0 μΜ, 26 h). The specimens are doubly stained with antibodies to acetylated tubulin (to identify daughter cells connected by a midbody) and counter-stained with TOPRO-3. Bar: 10 μm. **(B)** The same as above after staining with antibodies to aPKCζ (a marker of asymmetric division) and the pluripotency factors Nanog and Oct4. Bars: 10 μm. **(C)** Pattern of HP1α foci (constitutive heterochromatin) in newly divided cells, as identified by the presence of a midbody. The cells were maintained in the undifferentiated state in the presence of LIF or induced to differentiate for 2 days *in vitro* by removing LIF from the media, as indicated. Representative profiles of daughter cell nuclei are shown, together with box plots depicting the number and surface area of heterochromatic foci in each case. Bar: 10 μm. **(D)** Distribution of the lineage markers BraT (mesoderm) and Gata4 (endoderm) in daughter cell pairs at day 4 and day 8, respectively, of *in vitro* differentiation. The samples in these experiments were processed as described by Elabd *et al*. (see text) and counter-stained with TOPRO-3. (For additional explanations see text). “Mock” implies treatment with solvent alone (EtOH). Bar: 10 μm.

Despite the apparent malfunctioning of the error-correction mechanisms and the altered geometry of the mitotic spindle, dramatic changes in cell cycle kinetics and proliferation were not detected (Fig. 1D,E), indicating that the majority of the cells completed successfully mitosis. Consistent with this notion, newly divided cells possessed a normal-looking nuclear envelope, as could be confirmed by staining with anti-Lamin antibodies (Fig. 2A). Aberrant cytokinesis and formation of “micronuclei” were not observed, while cytoplasmic proteins (*e.g*., atypical protein kinase C zeta) and nuclear components (*e.g*., Nanog/Oct4) were distributed equally in the two daughter cells (Fig. 2B). From the sum of these results, it can be concluded that inhibition of Haspin kinase and elimination of the H3T3ph mark do not block symmetric, self-renewal divisions.

As could be judged by the distribution pattern of heterochromatin protein 1α (HP1α), treatment with 5-ITu did not affect the post-mitotic reassembly of heterochromatic foci in daughter cells. However, treatment with the Haspin inhibitor appeared to increase slightly the number of heterochromatic foci, with a corresponding decrease of their surface area. This effect could be detected both in self-renewing cells and in cells induced to differentiate in an adherent state for 48 hours (Fig. 2C).

To find out whether 5-ITu affects expression and post-mitotic distribution of cell lineage markers at later stages of *in vitro* differentiation, we followed the method of Elabd *et al*.^30^. Embryoid bodies (EBs), forming in “hanging drops”, were treated with 5-ITu and the constituent cells dissociated and seeded sparsely (as singlets). Examination of the specimens after one round of cell division (*i.e*., when the singlets became doublets) showed that newly divided cells contained the same amount of BraT (a mesodermal marker) and Gata4 (an endodermal marker), similarly to mock-treated controls (Fig. 2D). From these results it seems safe to conclude that inhibition of Haspin does not change the mode of cell division and does not impair initiation of lineage commitment.

### Effects on gene expression pattern

**Figure 3:**
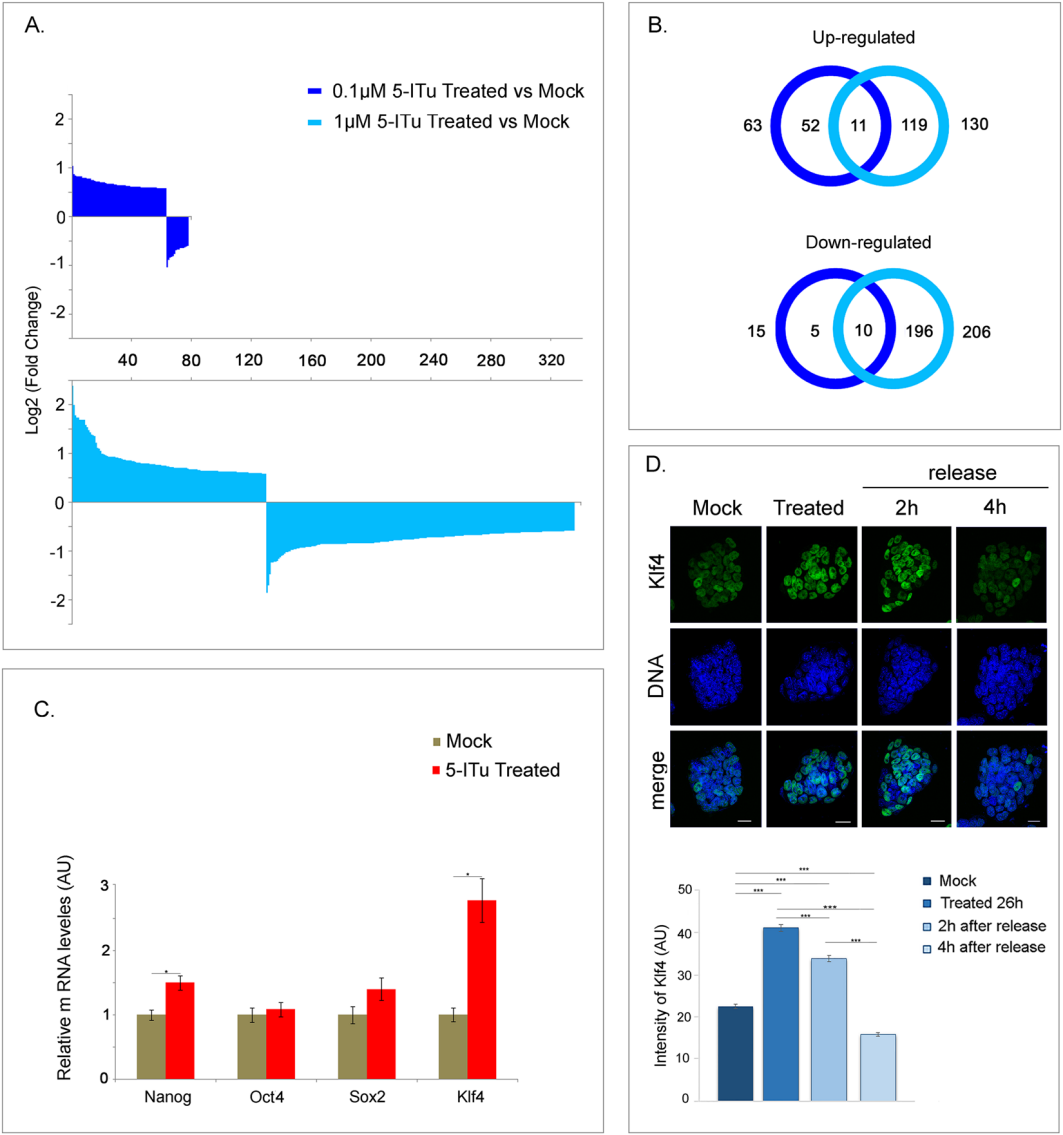
Effects of 5-ITu on gene expression. (**A**) Expression levels of 5-ITu-treated cells relative to mock-treated control (dark blue: 0.1 μΜ; light blue: 1.0 μΜ), as assessed by microarray analysis (log2 FC > 1.5, p value < 0.05). **(B**) Venn diagram showing a comparison of the affected genes in the two 5-ITu concentrations. **(C)** Expression levels of pluripotency markers Nanog, Oct4, Sox2 and Klf4 in 5-ITu-treated cells (1.0μΜ, 26 h), as quantified by qRT-PCR. SE bars are shown and one asterisk corresponds to p < 0.05. **(D**) Staining of 5-ITu treated cells (1.0μΜ, 26 h), before and after release, for Klf4. The samples are counter-stained with TOPRO-3. “Mock” implies treatment with solvent alone (EtOH) with the exception of microarray control, on which DMSO was used. A histogram shows the levels of Klf4 on a quantitative basis. Bar: 20 μm.

To examine more globally the effects of 5-ITu on E14 cells, we performed a genome-wide microarray screen. Αs shown in Fig. 3A, a significant number of genes were affected by the Haspin inhibitor. Sixty-three genes were upregulated and 15 downregulated >1.5 fold upon treatment with 0.1 μM 5-ITu for 26 h. On the other hand, 130 genes were upregulated and 206 downregulated when 5-ITu was used at 1.0 μM (Fig. 3B). Consistent with a bimodal mode of action, the genes affected at both concentrations of 5-ITu were relatively few, in comparison to the total number of affected genes in each condition (see Venn diagram).

Among the genes upregulated by 5-ITu were *Tdpoz2* and *Tdpoz3*, which encode factors involved in transcriptional regulation; several histone genes (*Hist4h4*, *Hist2h3b/3c2*, *Hist1h1e/4h*); *Hck*, which encodes a tyrosine protein kinase; *Tppp3*, which encodes proteins promoting tubulin polymerization; and the gene that encodes the pluripotency factor Klf4 (see Table in Fig. S3). The increase of Klf4 expression (approx. 1.7-fold) in cells treated with 1.0 μM 5-ITu could be confirmed by Real Time qRT-PCR, as depicted in Fig. 3C. Other pluripotency factors, such as Oct4 and Sox2, did not show expression differences, whereas Nanog expression was marginally affected. In line with the qPCR data, after immunostaining with specific antibodies, the intensity of the Klf4 signal appeared noticeably increased in 5-ITu-treated cells (Fig. 3D). However, 4 h after removal of 5-ITu from the medium, the levels of Klf4 returned to nearly normal levels, indicating reversibility.

**Figure 4:**
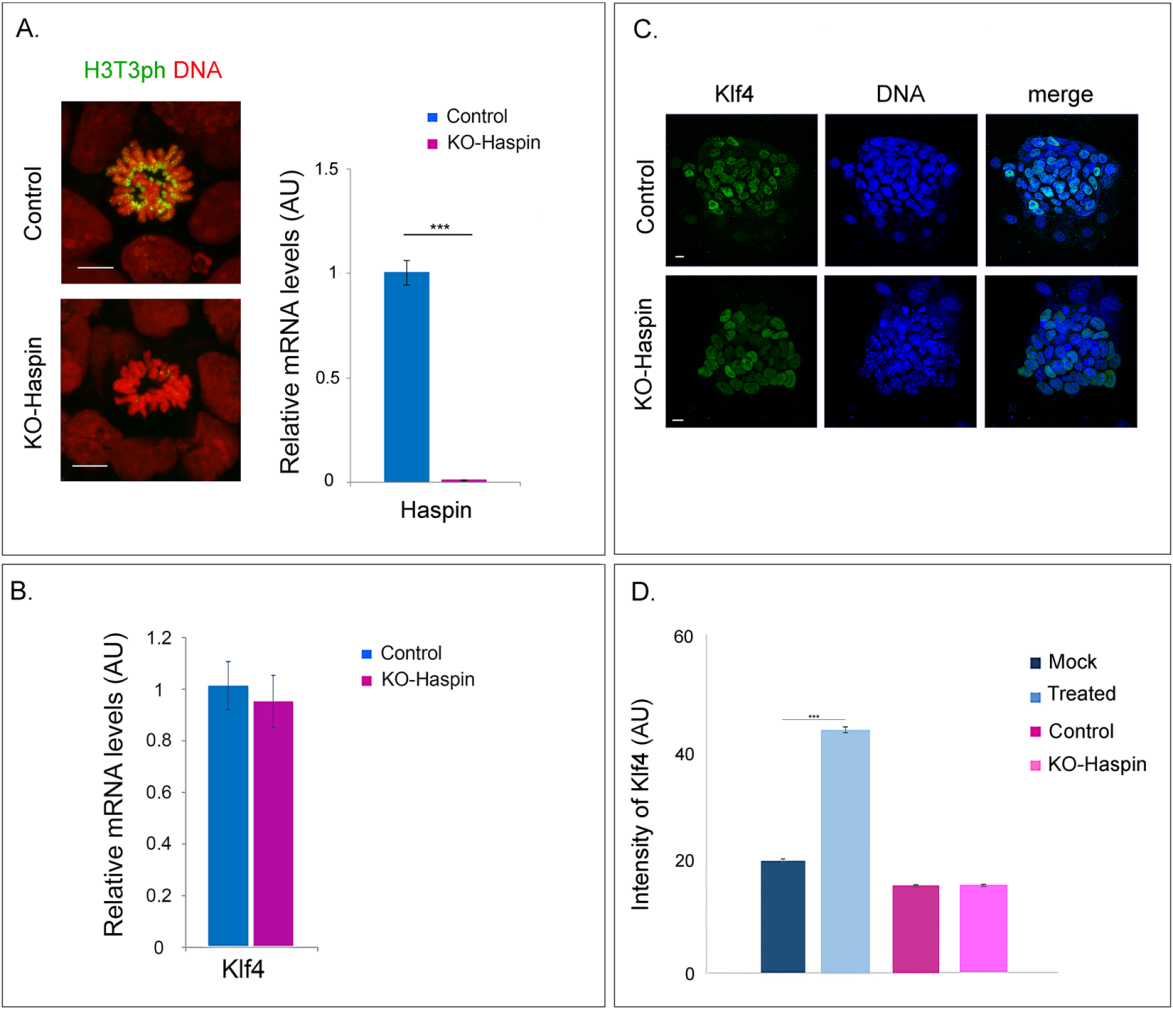
Expression of Klf4 in Haspin-knockout (KO) cells. (**A**) Staining of control and KO-Haspin cells for H3T3ph (left). The samples are counter-stained with PI. Bars: 5 μm. Expression levels of Haspin mRNA were measured by qRT-PCR (right). SE bars are shown and three asterisks correspond to p < 0.001. **(B)** Expression levels of Klf4 mRNA in control and KO-Haspin cells, as assessed by qRT-PCR. SE bars are shown. **(C)** Staining of control and KO-Haspin cells for Klf4. The samples are counter-stained with TOPRO-3. Bar: 10 μm. **(D)** Quantification of the results shown in (C). SE bars are shown and three asterisks correspond to p < 0.001 (see also Fig. S1A).

To determine whether Klf4 upregulation is due to the inhibition of the Haspin kinase, we constructed a stable cell line, in which the *Gsg2* gene was disrupted by Cas9-genome editing (Fig. S4). As shown in Fig. 4A, the H3T3ph signal in Haspin-knockout (KO) cells was eliminated. However, no change was observed in the expression levels of Klf4 by comparison to control cells (Fig. 4B–D). Confirming these observations, siRNA knockdown of Haspin in Hela cells did not increase the levels of Klf4. In fact, Haspin knockdown *decreased* Klf4 expression, contrasting the effect of 5-ITu in the same cell line (Fig. S5A). From these results it is clear that the upregulation of Klf4 by 5-ITu was apparently unrelated to Haspin inhibition.

To find out whether this “off-target” effect was context-dependent, we also examined the mouse myoblastic cell line C2C12, which expresses low levels of Klf4 in the proliferating state^31^. As shown in Fig. S5B, after treatment with 5-ITu the expression levels of Klf4 were slightly *decreased*. The sum of these observations indicates that 5-ITu does not interfere with vital or “housekeeping” cellular functions, but rather affects cellular processes that are unique to each cell type (see also “Discussion”).

### Effects on differentiation

**Figure 5:**
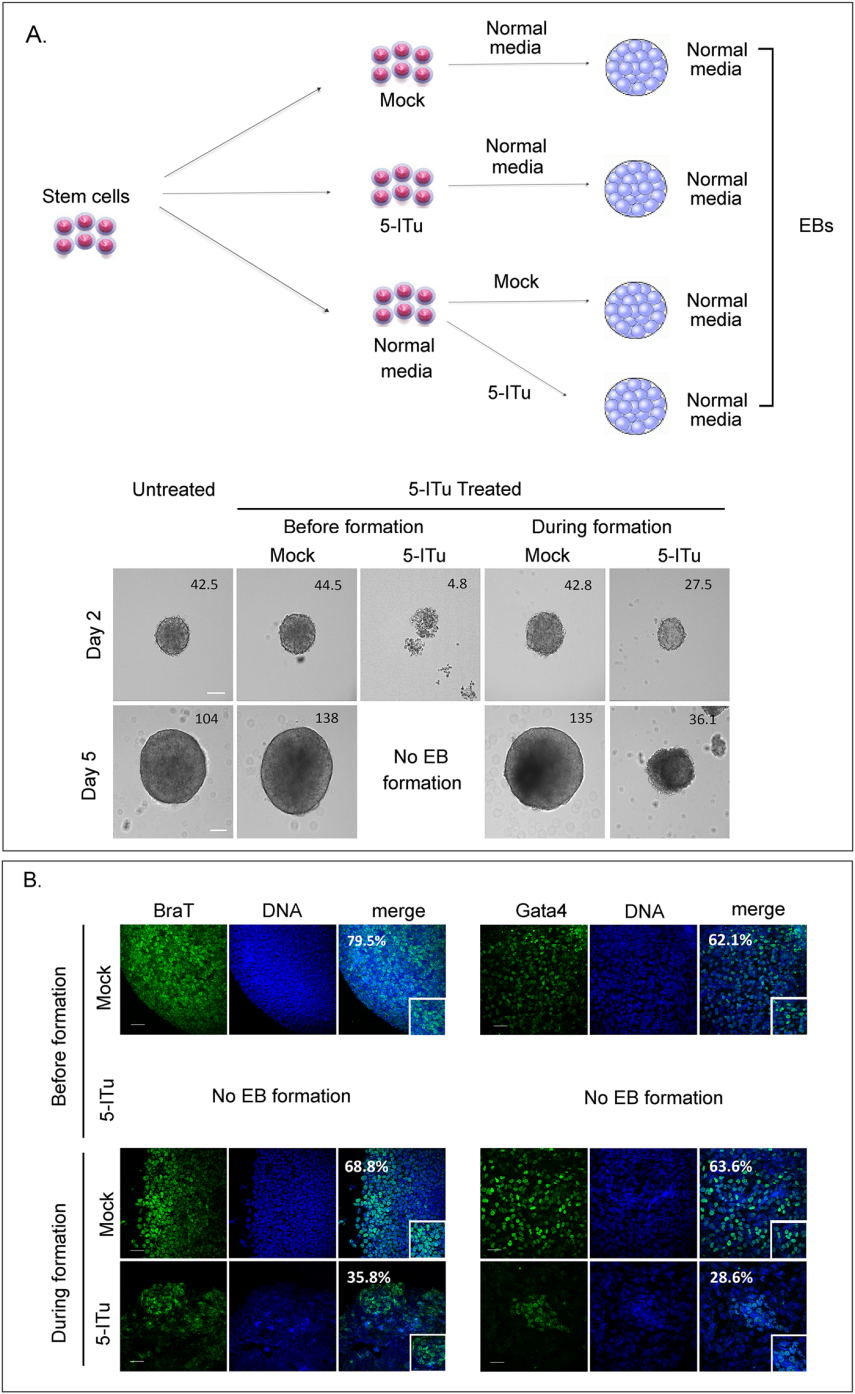
*In vitro* differentiation assays. (**A**) Differentiation schedule and representative profiles of embryoid bodies in each of the four experiments described in the text. The numbers correspond to the average surface area of embryoid bodies (in μm^2^). Bar: 50 μm. **(B)** Staining of embryoid bodies for the lineage-specific markers BraT and Gata4. The numbers on upper left represent the percentages of positive cells. The specimens are counter-stained with TOPRO-3. “Mock” implies treatment with solvent alone (EtOH). 5-ITu was used at 1 μΜ. Bar: 50 μm.

Comparing the results shown in Fig. 2D (*i.e*., the normal expression and distribution of lineage markers in 5-ITu treated cells) and the results shown in Fig. 3 (*i.e*., the transcriptional upsetting caused by the Haspin inhibitor), we considered that embryonic stem cells are affected by 5-ITu in a fashion that is not easily explainable. To investigate this problem further, we induced formation of EBs in four different ways, as outlined in the diagram of Fig. 5A. In the first case, the cells were mock-treated with the 5-ITu solvent alone (ethanol) before removal of LIF; in the second case, the cells were treated with 1.0μΜ 5-ITu for 26 h before removing LIF; in the third case, the cells were incubated with ethanol upon removal of LIF and during EB formation; and in the fourth case, the cells were exposed to 1.0 μΜ 5-ITu, upon LIF removal and during formation of EBs. In the end of this series, all samples were transferred in normal media and examined in the phase and the fluorescence microscope within a period of 48–96 h.

As shown in Fig. 5A (*images*), the mock-treated cells formed normal-looking EBs. On the other hand, whereas the cells treated *during in vitro* differentiation formed small EBs, the cells treated with 5-ITu *before* induction of differentiation did not form EBs at all. Interestingly, the “compromised” EBs produced in the former case contained a lower proportion of BraT- and Gata4-expressing cells than the controls (Fig. 5B).

**Figure 6:**
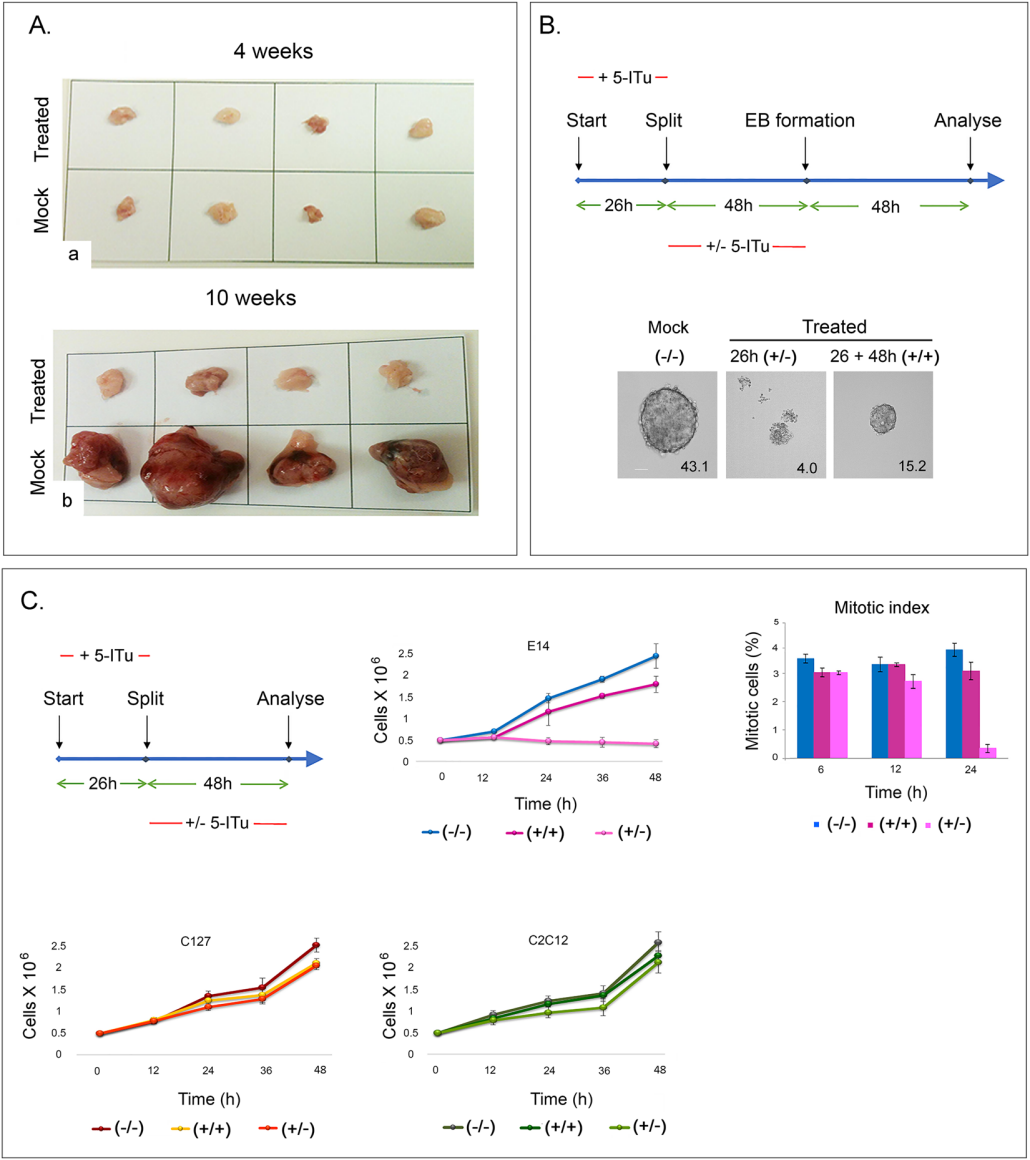
Teratoma formation and effects of 5-ITu on cell survival. (**A**) Tissue excised from mice injected with mock-treated or 5-ITu-treated cells at 4 (a) and 10 (b) weeks. **(B)** Schematic representation of 5-ITu treatment and *in vitro* differentiation. After a 26-hour treatment with 1.0 μM 5-ITu, the cells were split and either cultured with 5-ITu for another 48 h (+/+) or released into normal medium (+/−). Upon removal of LIF, all cells were suspended in “hanging drops” and induced to differentiate. Representative profiles of the embryoid bodies produced are shown. The numbers correspond to the average surface area of embryoid bodies (in μm^2^). Bar: 50 μm. **(C)** Schematic representation of the experiments done with undifferentiated cells. The panels depict growth curves of E14, C2C12 and C127 cells (the latter included here for a comparison). Histograms depicting the corresponding mitotic indexes are also shown (see also Fig. S1A).

To confirm the results obtained by *in vitro* differentiation assays, we tested whether the cells treated for 26 hours with 1μΜ 5-ITu could form teratomas. Four weeks after inoculation of untreated or treated E14 cells into immunodeficient mice, a cohort of animals were sacrificed to monitor potentially early lesions at the injection sites. As expected, all mice examined possessed scar tissue, indicating the absence of tumorous growth (Fig. 6A, *panel a*). However, 10 weeks post-inoculation, the mock-treated cells formed large teratomas, whereas the 5-ITu-treated cells did not (Fig. 6A, *panel b*). The teratomas formed by untreated E14 cells contained derivatives of all three germ layers (Fig. S6), assuring that the procedure was executed correctly. From the sum of these results, it can be concluded that exposure of E14 cells to 5-ITu prior to EB formation does not allow their proliferation and differentiation *in vivo*.

### Effects on cell cycle and growth

To better interpret the results obtained in differentiation assays, we incubated E14 cells for 26 hours with 5-ITu, split them, and continued culturing either in 5-ITu-free or in 5-ITu-containing media for another 48 hours. At the end of this incubation, we removed LIF and induced EB formation (Fig. 6B, *schematic diagram*). Normal-looking (but somewhat smaller) EBs formed when 5-ITu was continuously present. However, when 5-ITu was removed from the media (after the initial 26-hour incubation), EB formation was aborted (Fig. 6B, *images*). These results were highly reminiscent of what we had seen previously comparing the effects of 5-ITu in cells treated before or during *in vitro* differentiation (see Fig. 5A). Confirming these observations, cells released from 5-ITu inhibition exhibited a very low mitotic index and remained stationary. Such effects were not detected in other mouse cell lines, such as C127 or C2C12 (Fig. 6C), suggesting a context-dependent effect.

**Figure 7:**
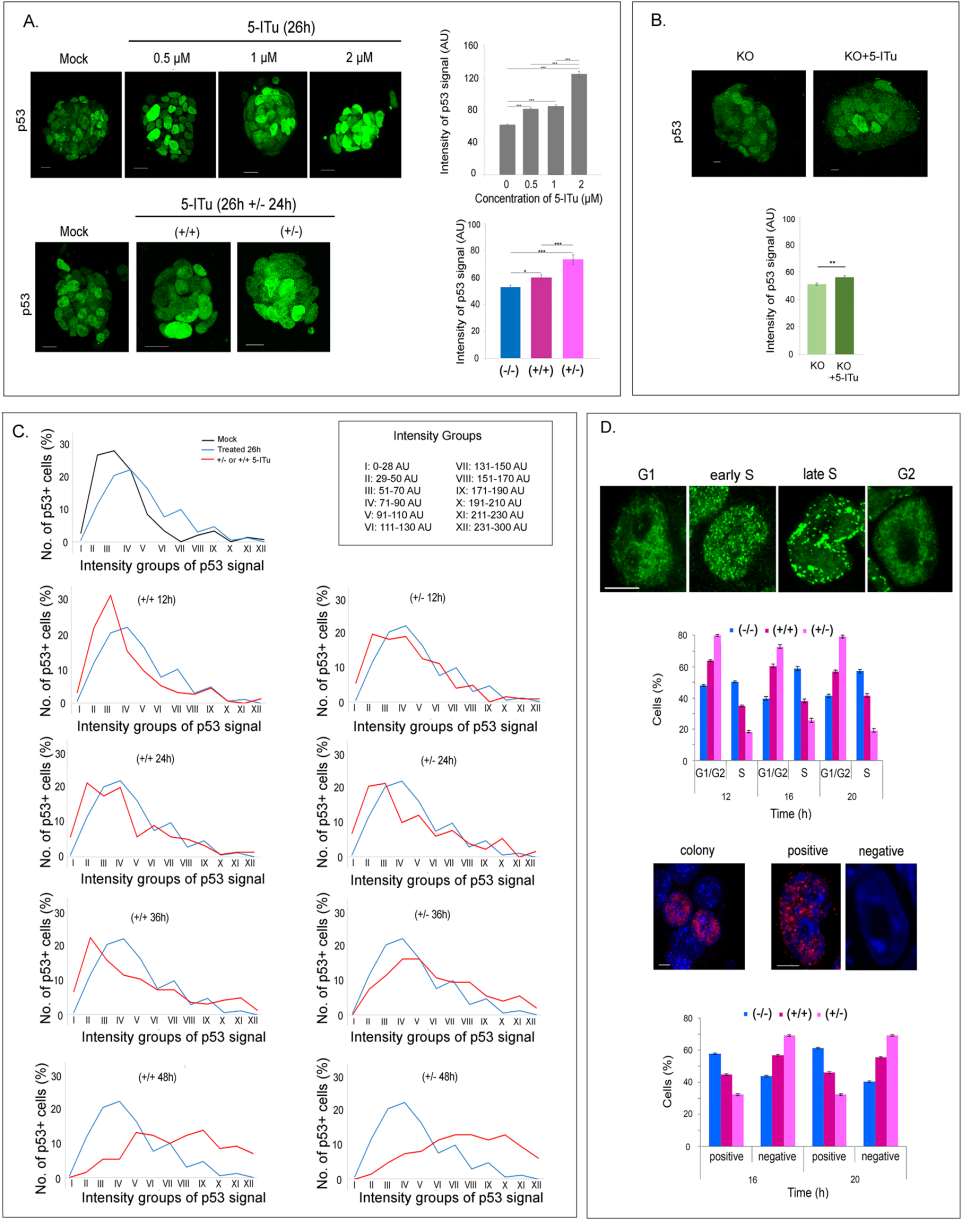
Effects of 5-ITu on p53 accumulation and cell cycle progression. (**A**) Top panel: accumulation of p53 in 5-ITu-treated cells (26 h). Representative profiles of E14 colonies after immunostaining for p53 and quantification of the signal intensities are shown. Bar: 20 μm. Lower panel: increase of p53 levels after release from 5-ITu (+/−) or continuation of 5-ITu treatment (+/+). Bar: 20 μm. (see also Fig. S1A). (**B**) Top panel: accumulation of p53 in 5-ITu-treated Haspin-KO cells (26 h). Representative profiles of colonies after immunostaining for p53 and quantification of the signal intensities are shown. Bar: 10 μm. Lower part: change in p53 levels after 26 h treatment of Haspin-KO cells with 5-ITu (see also Fig. S1A). **(C)** p53 levels across the cell population after release from 5-ITu (+/−) or continuation of 5-ITu treatment (+/+). The graphs depict the percentage of p53-positive cells in a 12-step signal intensity scale, as specified in the key. A time course 12–48 hours after the initial 5-ITu treatment is shown. (**D**) PCNA distribution and BrdU incorporation in cells processed as in (A). Bar: 5 μm. (see also Fig. S1A). 5-ITu was used at a concentration of 1 μΜ.

A previous report has claimed that 5-ITu induces p53 in mouse embryo fibroblasts^32^. In line with this observation, treatment of E14 cells with 0.5–2.0 μΜ 5-ITu caused a dose-dependent increase in p53 accumulation, as shown in Fig. 7A. Interestingly, the average levels of p53 were higher in the group that was released from the drug after an initial treatment than in the group of cells in which 5-ITu was continuously present. Comparing 5-ITu-treated and untreated KO cells, we have found that the drug causes a statistically significant increase in p53 levels (Fig. 7Β). However, this increase was less pronounced than the increase seen in wild type cells (see Fig. 7A,B), which indicates context influences. These data suggest that changes in p53 levels are unlikely to be related to Haspin inhibition, but is quite possible that Haspin-null cells have adapted to their Haspin-lacking status by downregulating p53 to more normal levels.

Assessment of p53 at 12-hr intervals revealed a differential response to 5-ITu. Up to 36 h, the cells released from 5-ITu contained few low-p53 figures. However, the population cultured in the continuous presence of 5-ITu contained a considerable proportion of low-p53 cells (Fig. 7B), suggesting some type of “resistance”. After 36 h, the proportion of cells containing medium/high amounts of p53 increased in both populations, indicating that “resistance” to 5-ITu was rather transient.

Knowing that p53 is activated, we finally checked if the release from 5-ITu was causing cell cycle arrest. We approached this problem in two independent manners. In one case, we monitored the incorporation of BrdU; in the other case, we stained the cells for PCNA, which gives a characteristic pattern in the G1/G2 and the S-phase (Fig. 7C). With both methods, we found that release from 5-ITu results in G1/S arrest, whereas the continuous presence of the inhibitor affects much less E14 cells. Possible interpretations of these results are discussed below.

## Discussion

Haspin is solely responsible for the establishment of H3T3ph during mitosis in somatic cells^13,14^. H3T3ph provides a binding site for Survivin and thus docks CPC at the inner centromere^15,16^. Although targeting of the CPC at the centromere is critical for correct chromosome alignment, at this point it is unclear whether Haspin-mediated phosphorylation of histone H3 is essential for cell division.

Recently obtained information suggests that Haspin’s function in mitosis might be redundant. As it turns out, CPC targeting to sites of microtubule-chromosome contact can be accomplished by Bub1-mediated phosphorylation of histone H2A at Thr120 (H2AT120ph). H2AT120ph creates a binding site for Sgo1, which then associates with the CPC component Borealin and deposits the complex to proximal centromere/kinetochore^33,34^. Furthermore, it has been recently reported that CPC can bind directly to nucleosomes *via* Borealin, which works upstream of Haspin-and Bub1-mediated centromere associations^35^. Consistent with functional redundancy, other studies have shown that mice with a disrupted *Gsg2* gene develop normally and exhibit a phenotype only in testes^36^, where Haspin was originally identified^6^. This might not be coincidental, because the H3T3ph mark plays a pivotal role in the germ stem cells (GPCs) of *Drosophila*, where it helps distinguishing pre-existing from newly synthesized histone H3^37^.

To examine the role of Haspin and H3T3ph during self-renewal and differentiation, we employed mouse embryonic stem cells and experimented with the Haspin inhibitor 5-ITu. In agreement to the published literature^15–17^, we found that sub-μM concentrations of this agent eliminate the H3T3ph mark and partially displace CPC from the centromere during mitosis. However, this does not appear to have severe consequences in the progress of mitosis and the entry of newly divided cells to G1, because both mock-treated and 5-ITu-treated cells divide symmetrically and continue self-renewing.

Despite the lack of overt defects, 5-ITu upsets the expression of several genes, including those that encode histone proteins and the pluripotency factor Klf4. Klf4 is upregulated by 5-ITu, which is worth noting, because, at the same time, p53 accumulates in the cell nucleus. p53 and Klf4 are known to act synergistically, activating p21/WAF, a cyclin-dependent kinase inhibitor^38,39^. Given this, it is not surprising that 5-ITu treated E14 cells show retarded growth and impaired entry into the S-phase. What is somewhat peculiar, however, is that removal of 5-ITu from the culture media increases the number of cells that are blocked in G1 and results in a complete halt of proliferation. A similar effect is observed upon induction of differentiation. If 5-ITu is continuously present, or added after initiation of *in vitro* differentiation, the cells can still form EBs. However, when the cells are (pre)treated with 5-ITu and then induced to differentiate in the absence of the drug, they fail to form EBs (or teratomas).

The observations reported here afford several interpretations. However, a key point, which should be taken seriously into account before attempting to interpret the data, is that p53 activation does not occur in Haspin-KO cells. Previous studies with mouse embryonic fibroblasts have shown that 5-ITu activates p53 and leads to cell cycle blockade^40^. Since this agent is essentially an adenosine analogue, it has been proposed that p53 activation is due to incorporation of a drug metabolite into DNA, which, then, results in double strand breaks. Invoking a similar mechanism, it would be easy to explain what happens in E14 cells: following DNA damage, p53 accumulates and induces expression of Klf4;^41^ then, Klf4 collaborates with p53 and induces expression of p21/WAF, delaying entry into the S-phase and, finally, causing cell death.

Although this mechanism is attractive in its simplicity, the genotoxic effects of 5-ITu and the cytostatic action of p53/Klf4 cannot be easily generalized. Since mouse C2C12 cells do not upregulate Klf4 and continue growing after 5-ITu treatment, it is unlikely that this agent acts universally by inducing base mis-incorporation and DNA damage. Pertinent to that is the fact that embryonic stem cells have a short G1 phase and known not to possess an identifiable G1/S checkpoint, because p21/WAF is under negative epigenetic control by histone deacetylase 1^41^. Although activation of p53 would allow transcription of the p21/WAF gene, the p21/WAF protein does not accumulate due to proteasome-dependent degradation^42^. In view of these data, it is unlikely that 5-ITu acts in embryonic stem cells in the same way as it acts in fibroblasts.

It could be argued that the latent effects of 5-ITu in E14 cells are the result of accumulating chromosome lesions, which develop when the H3T3ph mark is eliminated, the CPC mislocalized and the error-correction mechanism during mitosis disabled. As explained above, genomic damage/imbalance could induce p53, which, in turn, would induce Klf4 expression and (synergistic) activation of p21/WAF. However, this scenario is also unlikely for all the reasons explained above and for the additional fact that Haspin-KO cells do not overexpress Klf4 and possess normal levels of p53.

Examining the action of 5-ITu in E14 cells we need to consider that the drug has pleiotropic effects, besides Haspin inhibition. Apparently, 5-ITu treatment sets in motion opposing –or cross-inhibiting- processes, some of which are reversible and some not. For instance, although 5-ITu provokes sustained and non-reversible activation of p53, it might also cause reversible suppression of p21/WAF by enhancing histone deacetylation or protein degradation. Alternatively, cells treated for longer periods of time with 5-ITu might grow under “selection pressure”, which leads to establishment of suppressor mutations. One class of such mutations might cause inactivation of p21/WAF, thus altering the behavior of Klf4 from this of a suppressor to that of an enhancer of cell proliferation in a sub-population of cells, as has been shown by other authors^42,43^.

Obviously, more focused studies are required to characterize the non-Haspin targets of 5-ITu and identify the relevant compensatory mechanisms at the transcriptional and the post-transcriptional level.

## Materials and Methods

### Differentiation *in vitro*

E14 cells were induced to differentiate by the “hanging drop” method. The cells were cultured in Iscove’s Modified Dublecco’s Medium (IMDM) (Gibco), supplemented with 15% fetal bovine serum (FBS) (Biochrom AG), 2 mM penicillin/streptomycin (Gibco), 2mM L-glutamine (Gibco) and 450 mmol/L 1-Thioglycerol (M6145, SIGMA) in the absence of LIF. After counting, 20 μl drops with 500 cells each were placed on the lid of a 10 cm bacteriological dish, which contained tissue culture grade water at the bottom (for hydration). The drops were collected after 2 days and transferred to 35 mm dishes and cultured for another 2–4 days. For immunofluorescence assays, the day before the experiment, EBs were transferred into gelatine-coated dishes carrying coverslips.

### Teratoma formation

Cells were plated and incubated with 5-ITu (1.0 μM, 26 h) obtained from Santa Cruz Biotechnology (sc-3531A). The inhibitor was dissolved in EtOH or DMSO. Cells for each condition were trypsinized to produce single cells. One million cells were suspended in 0.1 ml of PBS and were injected subcutaneously into the flank of immunodeficient NOD-SCID gamma mice (NOD.Cg-Prkdcscid Il2rgtm1Wjl/SzJ) Approximately 4 weeks after the inoculation, 4 mice were sacrificed to study the progress of differentiation. The final timepoint of the control cohort was reached 10 weeks post inoculation. At that time another cohort of 4 animals was euthanized, the tumors were collected in 10% NBF, embedded in paraffin, and sectioned in the microtome. Sections of each teratoma were stained with Hematoxylin/Eosin and further analysed. All the experimental animals were housed in individually ventilated cages in the Animal House Facility of the Biomedical Research Foundation, Academy of Athens under pathogen-free conditions, in full compliance with the recommendations of Federation of Laboratory Animal Science Association. All procedures were approved by the institutional Bioethics Committee and the Greek Ministry of Agriculture (ref. number 6245) on the basis of the European Directive 86/609 concerning the protection of animals used for experimental purposes.

### Microarrays

Microarray experiments were carried out at the Genomics Core Facility of EMBL. Transcriptional analysis was performed using the Affymetrix GeneChip Mouse Gene 2.0 ST Arrays. Three biological replicates were used in each condition (DMSO only, 0.1 μM and 1.0 μM 5-ITu). Labelling and hybridization were carried out according to the standard Affymetrix protocol. Chips were scanned using the Hewlett-Packard GeneArray Scanner 3000 7 G. The quality and the background of the array data were assessed using the Expression Console (version 1.3) software (Affymetrix). Data analysis was carried out using the GeneSpring 13.0 software (Agilent Technologies). RNA normalization was performed and only probes above the background threshold (=353) were considered for further evaluation. Statistically significant transcripts were obtained based on their one-way Anova p value (<0.05). A cut-off on fold change >1.5 was selected. The data have been deposited in GEO database under the accession number GSE119737.

### Quantitative PCR

cDNA was prepared using the PrimeScript RT Reagent Kit (with gDNA Eraser-Perfect Real Time, Takara) according to instructions. Quantitative Real Time PCR reactions were carried out on a CFXConnect instrument (Biorad) using SYBR Fast Master Mix (2 × ) Universal (KAPA). Appropriate normalization genes (ATP5B and YWHAZ) were selected using the geNorm mouse reference kit (Primer Design) and data analysis was performed using the qbase^+^ analysis software (Biogazelle) according to MIQE guidelines. Statistical analysis was carried out using the nonparametric Mann Whitney test. The primer sequences used are tabulated here:

**Table:** 

Gene Name	Forward Primer	Reverse Primer
Nanog	CCAGTGGAGTATCCCAGCAT	GTTGGTCCAGGTCTGGTTGT
Oct4	TGGGCTAGAGAAGGATGTGG	TGGGAAAGGTGTCCCTGTAG
Klf4	GACTAACCGTTGGCGTGAGG	GTCTAGGTCCAGGAGGTCGT
Sox2	AAAGGGTTCTTGCTGGGTTT	AAACAAGACCACGAAAACGG
Haspin	ATGCTGAAAAGGTTTATGGGGA	GGCAGGATTTCCTCAAAGGTTT

### RNA interference

For silencing assays, the following siRNA and control sequences from Ambion were used: AM 1093, 141030, 242467 AM4642. The siRNAs were transfected into Hela cells at a concentration of 20 nM using the Lipofectamine RNAiMAX reagent (Invitrogen). Cells were analysed after 48–72 hours.

### CRISPR/Cas9-mediated gene deletion

The Haspin knock out clones were created using the Crispr/Double Nickase Cas9 system. More specifically, E14 cells were transfected either with two plasmids carrying the double Cas9 nickase and the Haspin sgRNAs (sc-420703-NIC, Santa Cruz Biotechnology) or with the crispr double nickase control plasmid (sc-437281, Santa Cruz Biotechnology). The gRNAs used to create the KO Haspin clone are shown in Fig. S4A. Transfections were carried out using the UltraCruz transfection Reagent (sc-395739, Santa Cruz Biotechnology). 24 hours after the transfection, 1.5 μg/ml puromycine was added to the medium. The antibiotic selection was kept for 72 hours. The cells were then left to recover and form new colonies for approximately 2 weeks. Newly formed single colonies were picked and transferred into separate wells of a 96-well plate. When they reached 80–90% confluency, they were trypsinized and transferred consecutively to a 24-well, a 12-well, a 6-well and, finally, to 60 mm plates.

### Amplicon Sequencing

Genomic DNA was isolated from the KO clone according to standard protocols. The region of interest was amplified using the following primers and Kapa HiFi hot start (Roche, KK2601).

**Table:** 

Primer	Sequence
For	CTCTTTCCCTACACGACGCTCTTCCGATCTTTCGAACGTACGCGGCGAGG
Rev	CTGGAGTTCAGACGTGTGCTCTTCCGATCTCTTCTGCGGGAGCCGAGGTC

The underlined sequences are handles, which are extended with adapter-barcoding sequences during a second PCR round. For indexing, the NEXTflex 16 S V1-V3 amplicon seq kit (Illumina Biooscientific, 4202–04) was used). Upon completion of the second PCR reaction, the size and yield of obtained amplicons was checked by agarose gel electrophoresis. Subsequently, amplicons were pooled together, purified using a PCR clean up column kit (Qiagen) and their concentration was measured on a Qubit 4 fluorimeter (ThermoFisher Scientific). Amplicons were sequenced bi-directionally on the MiSeq platform (Illumina), 250 bp from each side (plus 12 bp for barcodes), using the MiSeq Reagent Kit v2 (500cycle) (Illumina, MS-102–2003). Analysis was performed using the CRISPResso algorithm^44^. Based on amplicon sequencing data (file available upon request), both Haspin allelles were deleted. The deletion made by Cas9-editing allows for the transcription of a short 5’ sequence that corresponds to the cohesin-associated N-terminal fragment. This N-terminal peptide could not be identified immunochemically, because no antibodies recognizing this part of the Haspin molecule were available. Attempts to detect the corresponding RNA by RT-PCR were inconclusive. Therefore, whether this fragment is produced or not is ambiguous.

## Supplementary information

Supplementary information
