# Supplementary material for: Haspin-dependent and independent effects of the kinase inhibitor 5-Iodotubercidin on self-renewal and differentiation
Source: Sci Rep. 2020 Jan 14;10:232. doi: 10.1038/s41598-019-54350-4 (PMC6959359; doi:10.1038/s41598-019-54350-4)

Haspin-dependent and independent effects of the kinase inhibitor 5-Iodotubercidin on self-renewal and differentiation

Eleftheria Karanika[1,2], *Katerina Soupsana*[1,3], *Anastasia Christogianni*[2], *Dimitris Stellas,*[4,#], *Apostolos Klinakis*[4], *Anastasia S. Politou*[1,3] *and Spyros Georgatos*[1,2,*]

[1] Stem Cell and Chromatin Group, The Institute of Molecular Biology and Biotechnology, Biomedical Division, FORTH-ITE

The Laboratories of Biology[2] and Biological Chemistry[3], The University of Ioannina, Faculty of Medicine, 45 110 Ioannina, Greece

[4] Biomedical Research Foundation, Academy of Athens, Athens, Greece

[#] *Current address: Human Retrovirus Section, Vaccine Branch, Center for Cancer Research, National Cancer Institute at Frederick, 1050 Boyles Street, Building 535, Room 226A, Frederick, MD 21702-1201*

**Corresponding author: sgeorgat@cc.uoi.gr* Tel: 0030 26510 08044/07565

Supplementary materials and methods

Cell Culture

E14 cells (generously provided by Austin Smith's lab, Cambridge University, UK) were cultured in Glasgow Minimum Essential Medium (GMEM) (Gibco), supplemented with 15% fetal bovine serum (FBS) (Biochrom AG), 2mM penicillin/streptomycin (Gibco), 2mM L-glutamine (Gibco), 0.1mM non-essential aminoacids (Gibco), 1mM sodium pyruvate (Gibco), 0.1 mM β-mercaptoethanol (Sigma-Aldrich) and LIF (produced in house). Dishes were coated with 0.1% gelatine. Hela, C127 and C2C12 cells were cultured in Dulbecco's Modified Eagle's Medium with high glucose (DMEM) (Gibco), supplemented with 10% FBS (Biosera), 2mM penicillin-streptomycin (Biosera) and 2mM L-glutamine (Biosera).

Antibodies, indirect immunofluorescence and morphological assays

Primary antibodies included: anti-Nanog (ab80892, dilution 1:300), anti-Klf4 (ab129473, dilution 1:1000), anti-Aurora B (ab2254, dilution 1:1000), purchased from Abcam UK; anti-Oct3/4 (sc-5279, dilution 1:300), anti-Brachyury (N-19, sc-17743, dilution 1:50), anti-Gata4 (C-10, sc-1237, dilution 1:100), anti-p53 (FL-393, dilution 1:100) and anti-aPKCζ (dilution 1:200), obtained from Santa Cruz Biotechnology; anti-tubulin (Sigma-Aldrich, T5168, dilution 1:5000) and anti-HP1α (Millipore, MA3584, dilution 1:1000). Anti-PCNA staining (antibodies purchased from Santa Cruz and used at a dilution of 1:200), the cells were fixed with ice-cold methanol for 6 min. The antibodies for Lamin A (dilution 1:600), Lamin B (dilution 1:600), and anti-H3T3ph (dilution 1:750) were raised in house. For BrdU staining, the cells, after the required time of pulse with the thymidine analogue (obtained from Roche), were fixed with acid ethanol (90% ethanol, 5% acetic acid, 5% ddH2O) for 30min at RT.

Then, they were washed with PBS and incubated in 2M HCl with 0.2% Triton X-100 for 30 min at 37°C. HCl was neutralized using 0.1M sodium borate ($Na_2B_4O_7$) for 2min at RT. Cells were blocked using blocking buffer for 15min at RT. Subsequently, the samples were incubated with anti-BrdU overnight at 4°C. Secondary antibodies included: goat anti-rabbit 488 (Alexa, A11008), goat anti-rabbit 568 (Alexa, A11011), goat anti-mouse 568 (Alexa, A11004) and goat anti-human 568 (Alexa, A21090). For DNA staining TOPRO-3 (Invitrogen, T3605) or Propidium iodide were used. Indirect immunofluorescence was performed according to Maison et al., 1993. The cells were fixed with 1% or 4% formaldehyde, permeabilized with 0.2% Triton X-100 and "blocked" with 0.5% fish skin gelatin. A Leica SP5 confocal microscope was used to image the specimens. For comparisons, the same parameters (PMTs and offset) were employed for imaging and the images obtained were processed in the same manner. Fluorescence intensity and intensity plots were made using ImageJ.

RNA isolation

Total RNA from 3 biological replicates of each condition was isolated and purified using the RNeasy protect mini kit (Qiagen). All samples were subjected to an on column DNase digestion using the RNase-Free DNase set (Qiagen). RNA quality was assessed based on the Bioanalyzer values obtained (Agilent). Only samples with a RIN value above 9 were subsequently analyzed.

Statistics

All statistical analysis, unless stated otherwise, was performed using the SPSS statistical package. For more information on each experiment see Fig. S5.

Supplementary figure legends

Figure S1. Detailed data from statistical analysis for the data shown in the figures. **(A)** Number of experiments and cell counts for the data shown in each figure. **(B)** Statistical evaluation of the data in all experiments performed.

Figure S2. Effects of 5-ITu on the distribution of Aurora B in metaphase cells. The images show maximum projection (top) and individual z-sections of metaphase cells stained with anti-Aurora B antibodies and TOPRO3. Bar: 5μm. The histogram indicates the distribution of the Aurora B signal in relation to the centromere or the chromosome arms (see also Fig.S1A). 5-ITu was used at a concentration of 1μM.

Figure S3. List of selected genes affected by 5-ITu treatment. The fold change in expression levels is indicated.

Figure S4. (**A**). The gRNAs used to create the KO Haspin clone are depicted on the Haspin genome sequence. (Blue-20nt gRNAs, RED-PAM sequences). **(B).** The pie diagram shows the NHEJ (Non-Homologous end Joining) frequency of KO Haspin clone, as it was calculated by the CRISPResso algorithm. Sequence alignment reveals that the genomic sequence was altered in the Haspin KO clone due to a deletion of 10 nucleotides. Guide RNAs are framed. **(C).** Sequence alignment of the KO and the WT Haspin protein. Stop codon is highlighted in blue.

Figure S5. Effect of Haspin knockdown on Klf4 expression in Hela cells.

(A) Levels of Klf4 after 5-ITu treatment (1.0μM, 48h), as detected by indirect immunofluorescence. Bar: 20μm. Occurrence of H3T3ph in mitotic Hela cells after treatment with the Haspin inhibitor. Bar: 5μm. Levels of Klf4 after treatment of Hela cells with siRNA, as detected by indirect immunofluorescence. Bar: 20μm. Quantification of the results shown above. **(B)** Effect of 5-ITu (1μM) on Klf4 expression in C2C12 cells, as detected by indirect immunofluorescence. Bar: 20μm. Quantification of the presented results (see also Fig.S1A).

Figure S6. Teratoma formation. Anatomic sites of injection and histological analysis at 10 weeks. **(a)** Mice injected with untreated cells on one side and 5-ITu-treated (1μM) cells on the other. Arrow in the lower panel indicates scar tissue on the side injected with the latter. **(b)** Hematoxylin/Eosin staining of paraffin sections of the scar tissue shown in (a). Panels **(c-h)** Hematoxylin/Eosin staining of the teratoma. All three germ layers are present. Stratified squamous epithelium and elements of the epidermis are discernible in (c). Arrow indicates the formation of keratin pearls and squamous cells underneath (ectoderm). At the bottom of the section are glands (endoderm), while on the upper central part are masses of premature cartilage (mesoderm). Magnification: 5X. In **(d)**, simple tubular glands and cells of neuronal origin can be distinguished, consistent with neuroectodermal differentiation. Magnification: 10X. Formation of osteoid-premature bone is shown in **(e)**. Arrow indicates chondrocytes in an area that resembles hyaline cartilage. Magnification: 20X. Premature smooth muscle fibers and neural rosettes are detected in **(f)**. Magnification: 20X. Functional (secretory) epithelium is identified in (g). Also visible in this section is pseudostratified columnar ciliated epithelium. Arrow indicates the

ciliae. Magnification: 20X. Cartilage (purple formation on the bottom) and smooth muscle fibers (red formation on the left) are shown in **(h)**. Arrow indicates the formation of a mixed glandular-like structure. Magnification: 20X.

Supplementary references

Maison, C., Horstmann, H. & Georgatos, S.D., 1993. Regulated docking of nuclear membrane vesicles to vimentin filaments during mitosis. Journal of Cell Biology, 123(6 I), 1491–1505.

A.

Fig.1C	Cell No: Spindle length		Experiment No	Cell No: Misalignment			Experiment No
	Mock	Treated		Mock	Treated		
	141	115	3	138	130	Normal	4
				23	50	Misaligned	

Fig.2D	Mock	Treated	Mock/-LIF	Treated/-LIF	Experiment No
Surface area	340 foci	320 foci	209 foci	223 foci	3
No of foci	35 cells	27 cells	20 cells	21 cells	

Fig.4D	Cell No: Klf4 Intensity				Experiment No
	Mock	Treated	Control	KO-Haspin	
	2021	1975	2031	2241	4

Fig.6C	Cell No: Mitotic Index			Experiment No
	(-/-)	(+/+)	(+/-)	
6h	558	450	422	2
12h	480	550	300	
24h	502	380	430	

Fig.7A	Cell No: p53 Intensity: 5-ITu concentrations				Experiment No
	0µM	0.5µM	1µM	2µM	
	1122	559	579	317	3
	5-ITu Treated				
	(-/-)	(+/+)	(+/-)		
	239	299	185		2

Fig. 7B	Cell No: p53 Intensity: Haspin-KO		Experiment No
	Untreated	5-ITu	
	397	321	2

Fig.7D	Cell No: Brdu 16h positive	16h negative	20h positive	20h negative			Experiment No
(-/-)	375	285	410	270			2
(+/+)	198	252	181	219			
(+/-)	160	342	159	341			
	PCNA 12h G1/G2	12h S	16h G1/G2	16h S	20h G1/G2	20h S	
(-/-)	163	171	141	209	231	319	2
(+/+)	196	107	160	100	277	203	
(+/-)	260	60	270	105	418	102	

Fig.S2	Cell No: Aurora localization		Experiment No
	Mock	Treated	
	146	158	3

Fig.S5	Cell No: Klf4 Intensity Hela				Experiment No	Cell No: Klf4 Intensity C2C12		Experiment No
	Mock	Treated	Ctrl-siRNA	siRNA Haspin		Mock	Treated	
	1106	1152	1439	2124	2	1240	1425	2

B.

Experiment	p Value	Statistics	Cell Type
Spindle Length Fig.1C	9.45E-15	Mann-Whitney test	Treated vs Untreated
No of foci Fig.2C	1.23E-03	One-way Anova (1.7213E-14), Levene <0.05, Welch <0.05 with Tukey post hoc	E14 vs Inh
	7.42E-07		E14 vs -LIF/-Inh
	5.56E-13		E14 vs -LIF/+Inh
	1.66E-01		Inh vs -LIF/-Inh
	1.00E-06		Inh vs -LIF/+Inh
	8.75E-03		-LIF/-Inh vs -LIF/+Inh
Surface Area of foci Fig.2C	3.09E-13	One-way Anova (8.3532E-71), Levene <0.05, Welch <0.05 with Tukey post hoc	E14 vs Inh
	2.27E-13		E14 vs -LIF/-Inh
	2.27E-13		E14 vs -LIF/+Inh
	1.68E-12		Inh vs -LIF/-Inh
	2.27E-13		Inh vs -LIF/+Inh
	5.49E-02		-LIF/-Inh vs -LIF/+Inh
KLF4 release Fig.3D	1.33E-12	One-way Anova (4.5523E-181), Levene <0.05, Welch <0.05 with Tukey post hoc	Control vs 26hrs
	1.33E-12		Control vs 2hrs
	1.39E-12		Control vs 4hrs
	1.37E-12		26hrs vs 2hrs
	1.33E-12		26hrs vs 4hrs
	1.33E-12		2hrs vs 4hrs
Intensity KLF4 Fig.4D	1.89E-213	Mann-Whitney test	Mock vs Treated
	4.19E-01	Mann-Whitney test	Control vs KO
Intensity p53 Fig7A	5.24E-13	One-way Anova (3.3054E-109), Levene <0.05, Welch <0.05 with Tukey post hoc	Control vs 0.5uM
	5.24E-13		Control vs 1uM
	5.24E-13		Control vs 2uM
	3.61E-01		0.5uM vs 1uM
	5.24E-13		0.5uM vs 2uM
	5.24E-13		1uM vs 2uM
Intensity p53 Fig.7A	4.40E-02	One-way Anova (1.7772E-7), Levene <0.05, Welch <0.05 with Tukey post hoc	Control vs ++
	8.64E-08		Control vs +-
	7.95E-04		++ vs +-
Intensity p53 Fig.7B	0.00267	Mann-Whitney test	KO vs KO-Inh
Intensity KLF4 Fig.S5A	1.97E-11	Mann-Whitney test	Mock vs Treated
	2.99E-107	Mann-Whitney test	Control siRNA vs siRNA
Intensity KLF4 Fig.S5B	3.00E-06	Mann-Whitney test	Mock vs Treated

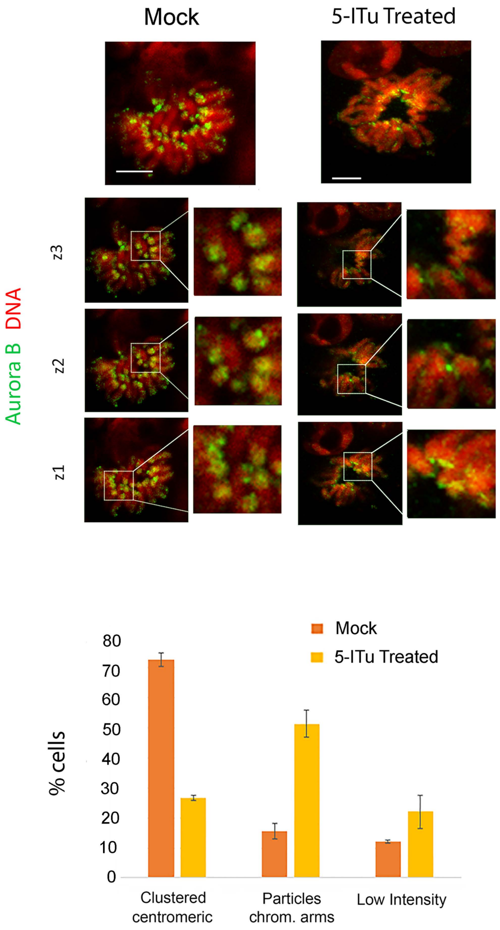
Mock
5-ITu Treated
Aurora B DNA
z3
z2
z1
% cells
80
70
60
50
40
30
20
10
0
Mock
5-ITu Treated
Clustered centromeric
Particles chrom. arms
Low Intensity

0.1µM Vs Control		
**Known genes**	**FC (abs)**	**Regulation**
AU015836	2.05	up
Hck	1.82	up
Prss42	1.78	up
Pdgfc	1.76	up
Hist2h3c2	1.72	up
Ttll6	1.65	up
Cd68	1.65	up
Cbr3	1.62	up
Glp1r	1.60	up
Ccnd2	1.59	up
Cyp2b23	2.04	down
AF357355	1.60	down
Ror1	1.58	down

1µM Vs Control		
**Known genes**	**FC (abs)**	**Regulation**
Tdpoz3	3.97	up
Hist4h4	3.45	up
Hist2h3b	3.33	up
Hist1h1e	2.99	up
Tdpoz2	2.69	up
Prss42	2.34	up
Hist2h3b	1.92	up
Hist1h4h	1.91	up
Cd68	1.88	up
Tppp3	1.8	up
Hist1h12ao	1.74	up
**Klf4**	**1.7**	**up**
Ammerc1	3.24	down
Lefty1	2.34	down
Chac1	2.18	down
Cdkn3	1.7	down

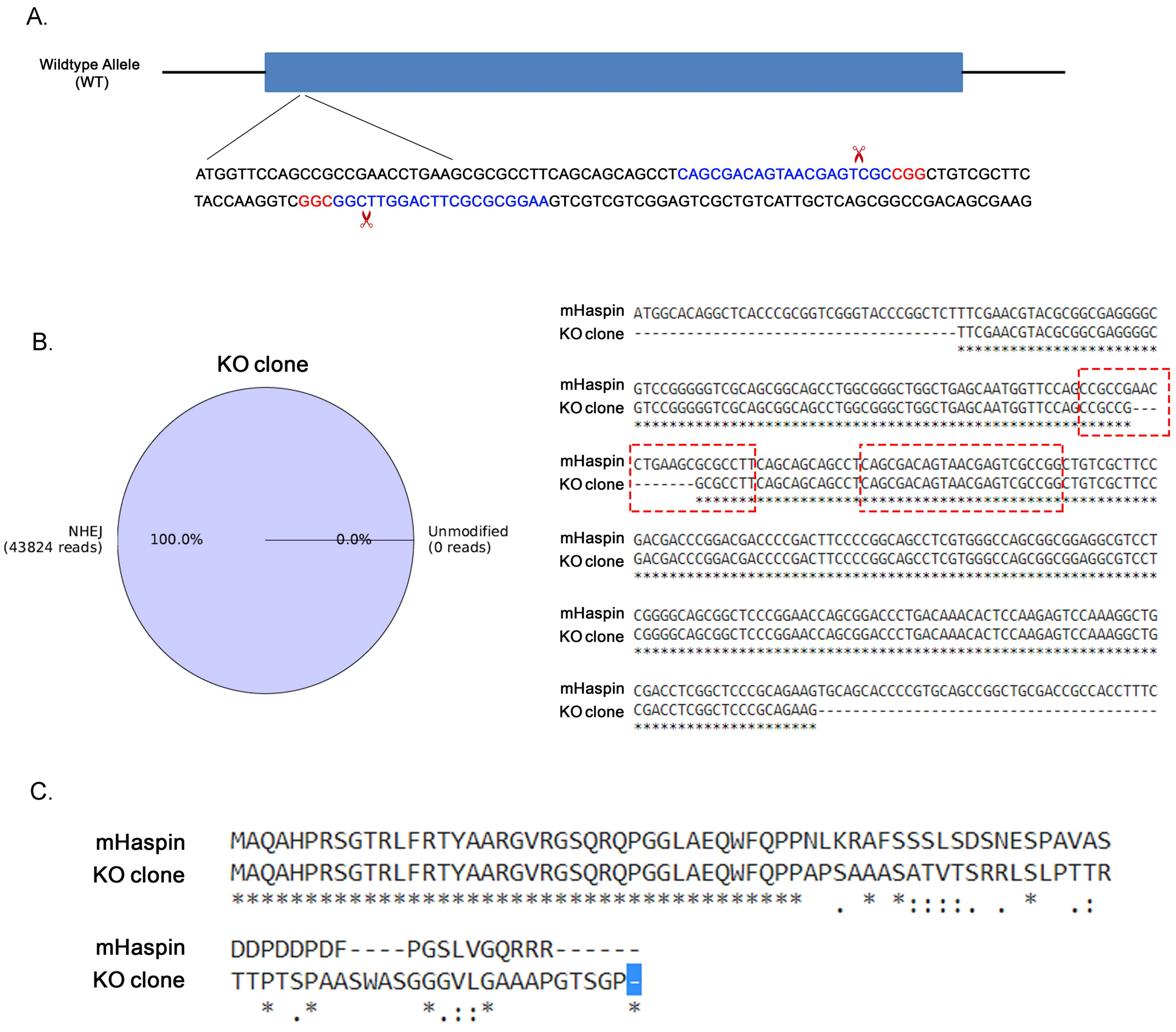
A.
Wildtype Allele
(WT)
ATGGTTCCAGCCGCCGAACCTGAAGCGCGCCTTCAGCAGCAGCCTCAGCGACAGTAACGAGTCGCCGGCTGTCGCTTC
TACCAAGGTCGGCGGCTTGGACTTCGCGCGGAAGTCGTCGTCGGAGTCGCTGTCATTGCTCAGCGGCCGACAGCGAAG
B.
KO clone
NHEJ
(43824 reads)
100.0%
0.0%
Unmodified
(0 reads)
mHaspin ATGGCACAGGCTCACCCGCGGTCGGGTACCCGGCTCTTTCGAACGTACGCGGCGAGGGGC
KO clone -----------------------------------TTCGAACGTACGCGGCGAGGGGC
mHaspin GTCCGGGGGTCGCAGCGGCAGCCTGGCGGGCTGGCTGAGCAATGGTTCCAGCCGCCGAAC
KO clone GTCCGGGGGTCGCAGCGGCAGCCTGGCGGGCTGGCTGAGCAATGGTTCCAGCCGCCG---
mHaspin CTGAAGCGCGCCTTCAGCAGCAGCCTCAGCGACAGTAACGAGTCGCCGGCTGTCGCTTCC
KO clone -------GCGCCTTCAGCAGCAGCCTCAGCGACAGTAACGAGTCGCCGGCTGTCGCTTCC
mHaspin GACGACCCGGACGACCCCGACTTCCCCGGCAGCCTCGTGGGCCAGCGGCGGAGGCGTCCT
KO clone GACGACCCGGACGACCCCGACTTCCCCGGCAGCCTCGTGGGCCAGCGGCGGAGGCGTCCT
mHaspin CGGGGCAGCGGCTCCCGGAACCAGCGGACCCTGACAAACACTCCAAGAGTCCAAAGGCTG
KO clone CGGGGCAGCGGCTCCCGGAACCAGCGGACCCTGACAAACACTCCAAGAGTCCAAAGGCTG
mHaspin CGACCTCGGCTCCCGCAGAAGTGCAGCACCCCGTGCAGCCGGCTGCGACCGCCACCTTTC
KO clone CGACCTCGGCTCCCGCAGAAG---------------------------------------
C.
mHaspin MAQAHPRSGTRLFRTYAARGVRGSQRQPGGLAEQWFQPPNLKRAFSSSLSDSNESPAVAS
KO clone MAQAHPRSGTRLFRTYAARGVRGSQRQPGGLAEQWFQPPAPSAAASATVTSRRLSLPTTR
mHaspin DDPDDPDF----PGSLVGQRRR------
KO clone TTPTSPAASWASGGGVLGAAAPGTSGP-

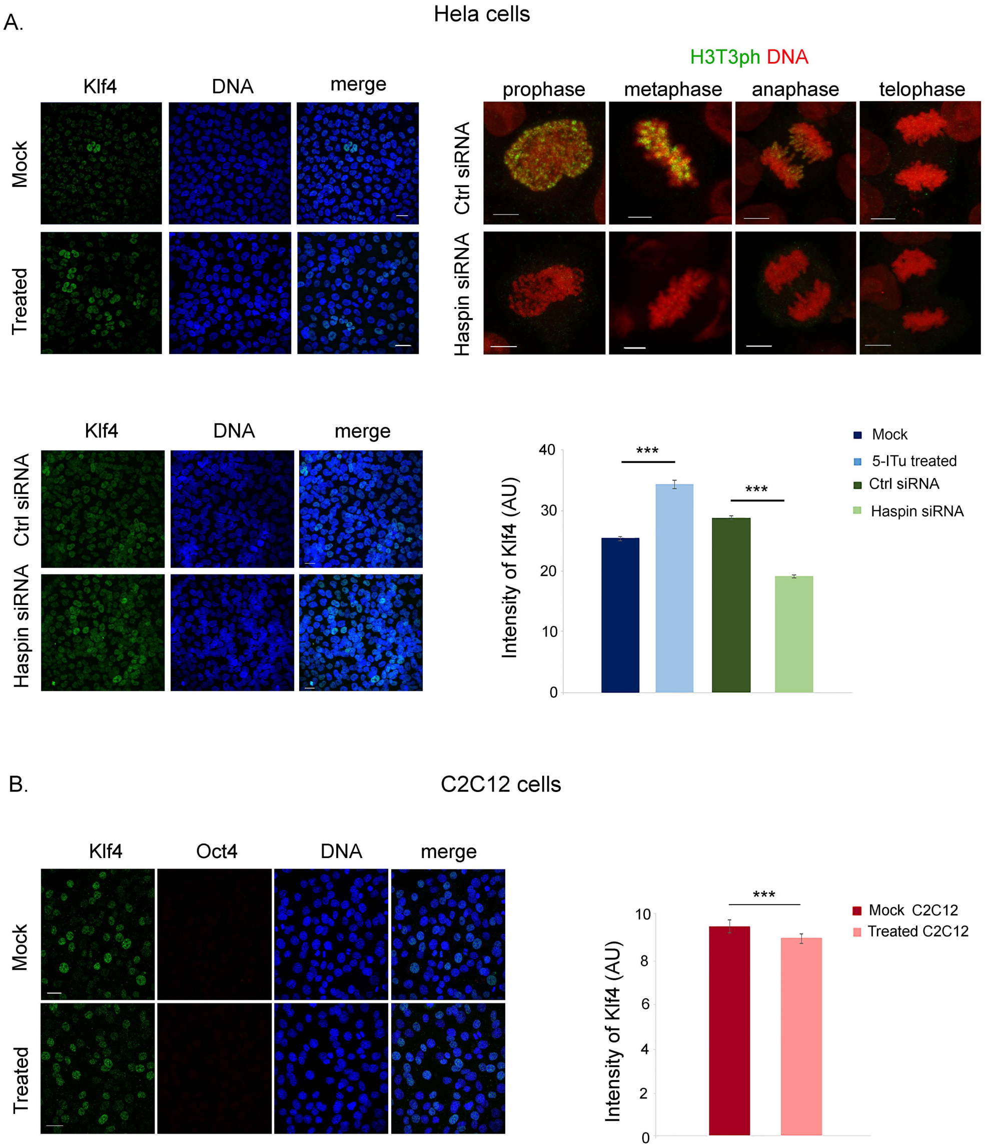
A.
Hela cells
Klf4
DNA
merge
Mock
Treated
H3T3ph DNA
prophase
metaphase
anaphase
telophase
Ctrl siRNA
Haspin siRNA
Klf4
DNA
merge
Ctrl siRNA
Haspin siRNA
Intensity of Klf4 (AU)
0
10
20
30
40
***
***
Mock
5-ITu treated
Ctrl siRNA
Haspin siRNA
B.
C2C12 cells
Klf4
Oct4
DNA
merge
Mock
Treated
Intensity of Klf4 (AU)
0
2
4
6
8
10
***
Mock C2C12
Treated C2C12

Supp. fig 6

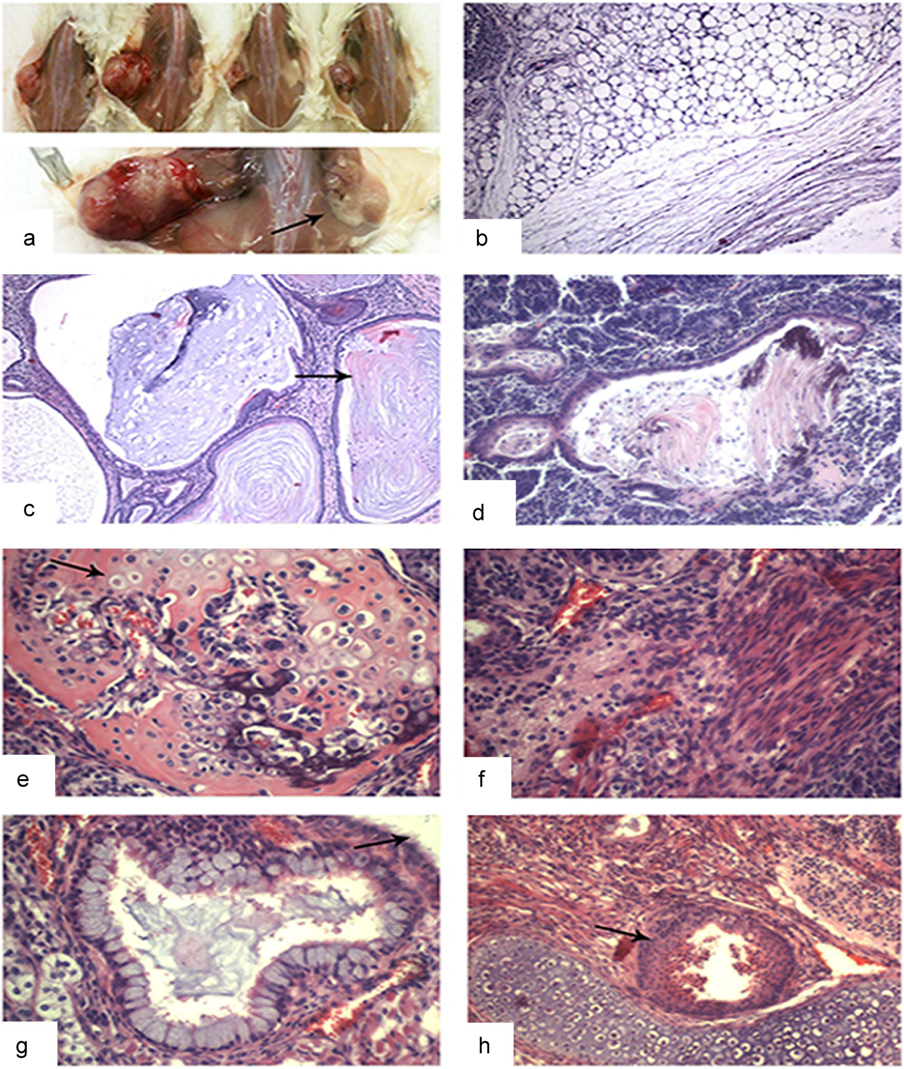

Supplement: Supplementary file 1 — Supplementary information [file 41598_2019_54350_MOESM1_ESM.pdf]
